# Premenstrual Symptoms and Work: Exploring Female Staff Experiences and Recommendations for Workplaces

**DOI:** 10.3390/ijerph18073647

**Published:** 2021-03-31

**Authors:** Claire Hardy, Myra S. Hunter

**Affiliations:** 1 Division of Health Research, Faculty of Health and Medicine, Lancaster University, Lancaster LA1 4AT, UK; 2 Health Psychology Section, Department of Psychology, Institute of Psychiatry, Psychology and Neuroscience, King’s College London, London SE1 9RT, UK; myra.hunter@kcl.ac.uk

**Keywords:** premenstrual symptoms, severity, PMS/PMDD, work, disclosure, online survey

## Abstract

Most women experience some premenstrual symptoms during their reproductive years. Yet, this is an under-researched health issue, particularly in the context of work. This study aimed to: (i) understand the prevalence and severity of premenstrual symptoms experienced by working females, and their association with key work outcomes; (ii) explore factors that may be influencing these symptoms and their severity; and (iii) examine how organizations might help staff with premenstrual symptoms that may be impacting their working lives. An online, anonymous survey collected quantitative and qualitative data from 125 working women in the UK. Over 90% of the sample reported some premenstrual symptoms; 40% experienced premenstrual symptoms moderately or severely. Higher symptom severity was significantly (*p* < 0.05) associated with poor presenteeism, intention to reduce working hours, and higher work absence (time off work, being late, leaving early). Moderate/severe symptoms were significantly associated with several individual-related variables: lower perceived general health, higher alcohol consumption, poorer sleep quality, anxiety, depression, hormonal contraception, and using fewer coping approaches towards premenstrual symptoms (avoiding harm, adjusting energy levels); and work-related variables: poorer work–life balance, lower levels of psychological resilience, higher perceived work demands, less control over work. Disclosure of premenstrual symptoms and sickness absence because of premenstrual symptoms was very low, typically because of perceptions of appropriateness as a reason for work absence, gender of line managers (male), and it being a personal or embarrassing topic. Staff with moderate to severe premenstrual symptoms were statistically more likely to disclose reason for absence than those with milder symptoms. Recommendations and suggestions for employers and line managers include the need to train staff to improve knowledge about women’s experience of premenstrual symptoms, to be able to communicate effectively with women and to provide tailored support and resources for those who need it. Implications for future research, policy and practice are discussed.

## 1. Introduction

Menstruation is a naturally occurring phenomenon for women. Typically experienced between the ages of 13–51 years in Western cultures [1], menstruation is commonly preceded by some bodily sensations or symptoms, for up to 90% of women [2]. These are often described as either psychological, such as anxiety, depression, mood swings, or physical, including bloating, headaches, breast tenderness, which typically last between 7–14 days before stopping when menses begins [3]. The duration, frequency, and severity of symptoms varies between women [4]. Some women may experience little of no disruption whilst others experience premenstrual symptoms that negatively impair normal functioning whilst symptoms are present. They can vary from month to month for each individual but typically a pattern is experienced [3].

Two conditions typically associated with severe premenstrual symptoms are Premenstrual Syndrome (PMS) and a more severe form of PMS called Premenstrual Dysphoric Disorder (PMDD). Both reflect the cyclical presentation of multiple symptoms that are severe enough to disrupt everyday life. PMS typically involves moderate to severe presentation of at least two symptoms (one physical and one psychological) and PMDD involving five or more symptoms that impact on normal functioning during this time in the menstrual cycle only (also with specific symptom type presentation; see the World Health Organization’s International Classification of Disorders (ICD) [5] and American Psychological Association’s Diagnostic and Statistical Manual of Mental Disorders (DSM-V) [6] for more specific diagnostic information). Around 40–50% of females may experience premenstrual symptoms regularly and severely enough to receive a diagnosis of PMS [2,7] and around 5% may have PMDD [4]. Estimates have changed overtime and vary depending on the measurements and criteria used [8]. However, use of terms such as “PMS” and “PMDD” has been criticized for pathologizing a normal part of women’s experience, and also because someone may experience one symptom severely enough to impact on daily life, yet not receive a diagnosis nor support (e.g., [9,10]).

Nevertheless, the impact of premenstrual symptoms, however, can be considerable for women, their families, and society [11]. For example, women may experience around 3000 days of difficult or problematic premenstrual symptoms over the course of their reproductive years [12].

### 1.1. Premenstrual Symptoms and Work

A growing body of evidence is highlighting the importance of addressing premenstrual health in the work context. To date, research has primarily focused on the relationship between premenstrual symptom severity and the rates of absenteeism, job performance and productivity. Several studies have shown reductions in work productivity, performance, and higher absence from work in staff who reported problematic premenstrual symptoms (e.g., [13,14,15,16,17,18,19,20]), with very few exceptions (e.g., [21]). Borenstein and colleagues [13] found a 13.7% absenteeism rate (days taken as absence) for women with premenstrual symptoms and a 15% reduction in work productivity (*N* = 364). Chawla [14] found taking half days off work were more common than taking full days of absence in women with premenstrual symptoms.

Borenstein and colleagues [13] estimated the indirect costs associated with such work impairments in the USA to be an increase of $4333 per individual per year compared to those without PMS. However, rates of absence were believed to be under-estimated due to the taboo nature of the health issue, i.e., staff may have felt too embarrassed to report premenstrual symptom difficulty and may have given other reasons for their absence leading to a lower number of reported cases.

According Gatrell and colleagues [22], women’s bodies being ‘unstable and uncontrollable’ are often viewed unfavorably compared to men’s in organizational contexts-being affected by menstruation, pregnancy, motherhood or non-motherhood, and the menopause. Women may be aware of negative attitudes at work and therefore decide not to draw attention to female-specific experiences related to their rebodies. Lack of disclosure, as well as fears around disclosing premenstrual related difficulties at work, has been found in a one qualitative study of women with a diagnosis of PMDD [17]. For example, some women with PMDD did not disclose this to their line managers for fear of a negative response or being stigmatized if they confided in them or work colleagues. In instances when disclosure did occur, a mixture of positive and negative responses was reported. The women expressed a need for more awareness, understanding and support within the workplace. Further exploration of negative attitudes and disclosure is needed as some staff may be experiencing difficulties but not getting any help or support from their workplace.

The potential impact of premenstrual symptoms on work outcomes is an important research question. Although under-researched, there appears to be some consistency in supporting the view that impairment of performance and/or productivity and absenteeism rates may be reported by women with moderate or severe premenstrual symptoms. What we do not yet fully understand is the possible influence of premenstrual experiences on other work outcomes, such as presenteeism (i.e., being at work when not 100%), and intention to leave the organization or working altogether [17,23]. In the study by Hardy and Hardie [17], levels of presenteeism (i.e., being at work when not 100% because of their premenstrual symptoms) were evident for staff with severe premenstrual symptoms/PMDD, as were impaired job performance and needing to take time off work. Over time, the women in the study reported that the cyclical pattern of premenstrual symptoms and their impact on their working life affected their work life balance, as staff attempted to compensate for their impaired time at work of absence by working longer hours. Ultimately, this led to some of these women leaving their jobs or being asked to leave and negatively impacted on the woman’s career, which some said they had to give up on altogether. Research is therefore needed to examine a wider range of work outcomes, as well as work-related factors that might be associated with premenstrual symptoms and their severity.

### 1.2. Aims and Research Questions

This paper aims to: (i) understand the prevalence and severity of premenstrual symptoms in working females and their association with key work outcomes; (ii) explore factors that may be influencing these symptoms and their severity; and (iii) examine how organizations might help staff whose working lives are impacted by premenstrual symptoms. Using cross-sectional data collected from a UK trade union’s women’s health survey, this paper poses the following research questions:

1. What is the prevalence and severity of premenstrual symptoms in a sample of working women in the UK?
2. To what extent does premenstrual symptom severity relate to work absenteeism, job performance, presenteeism, and turnover intentions?
3. Are there any individual or work-related variables that are significantly associated premenstrual symptom severity?
4. To what extent are premenstrual symptoms and their consequences on work being disclosed to line managers and why?
5. What recommendations and suggestions do staff have for employing organizations and line managers to support and help staff with work difficulties associated with premenstrual symptoms?

## 2. Materials and Methods

Female members of a UK trade union and professional association for family court and probation staff in England, Wales and Northern Ireland were invited to complete an anonymous electronic survey. The trade union were interested in working with academics to understand the menopause and premenstrual experiences of their members. The survey was created by the authors to investigate the experience, attitudes, and recommendations of female members of these reproductive health topics. The survey collected qualitative and quantitative data.

Female members were invited to complete the anonymous electronic survey via an email that was distributed from an internal contact within the trade union. The email contained a brief description of the study, the researchers’ contact details, and the link to the survey. There were approximately 500 female members across six geographical areas that could be contacted via their email address at the time of the survey.

The survey was hosted on the platform SurveyMonkey and took approximately 20 min to complete. Before participants completed the survey, they were presented with details of the purpose and scope of the study. Participants were informed that the survey was anonymous and voluntary. They were invited to click a consent box before being allowed to complete the survey questions. The survey had to be completed in one sitting and could be done using a desktop, tablet, or mobile phone device. It was first piloted with five people, including three women from within the trade union and two academic researchers with knowledge and expertise in survey design. This was done to ensure language, content, and length were acceptable before conducting the main data collection.

Ethical approval for this study was granted from the Ethical Review Committee at King’s College London (reference number: HR-15/16-2492). Feedback on the results was provided in both an internal summary feedback report for the union and presented at their annual Women’s Conference.

### 2.1. Measures

Self-report data was collected from participants classified as premenopausal (i.e., only respondents who were having regular menstrual periods for them) and were not pregnant. The survey included questions from pre-existing measures and bespoke items to gather quantitative and qualitative data of interest for the trade union member survey.

#### 2.1.1. Individual and Work-Related Variables

Demographic and health-related questions included (see Supplementary Table S1 for all response options): *age, ethnicity, educational level, sexuality, relationship status*, and whether they had *dependents/caring responsibilities*. Health and wellbeing questions included: *general health* (5-point scale, 1 (poor) to 5 (excellent)) [24], *body mass index* (BMI), weekly *exercise frequency* (1 (rarely/never) to 6 (every day), weekly *alcohol intake* (1 (none), 2 (1–6 units per week), 3 (7–13 units per week), 4 (+14 units per week)), *smoking status* (never, ex-smoker, or smoker), *sleep quality* (4-point scale item, 1 (very good) to 4 (very bad), from the Pittsburgh Sleep Quality Index (PSQI) [25]), levels of *depression* (7 items using a 4-point scale: 1 (not at all), to 4 (nearly every day), example item “Feeling nervous, anxious or on edge”, α = 0.92, GAD-7, [26]) and *anxiety* (9 items using 4-point scale: 1 (not at all), 2 (several days), 3 (more than half the days), 4 (nearly every day), example item “little interest or pleasure in doing things”, α = 0.89, PHQ-9, [27]).

Work-related variables included (see Supplementary Table S2 for all response options): weekly *working hours*, *work status*, *work patterns/shift work*, *flexible working*, *type of work*, *managerial/supervisory responsibilities*, and the *sector* in which they worked. Perceptions of work questions included: *job satisfaction* (single item, 7-point scale: 1 (extremely dissatisfied) to 7(extremely satisfied) [28]), *job stress* (4-point scale: 1 (not stressful) to 4 (extremely stressful) [29]), *work–life balance* (3 items, example item “I manage to balance the demands of my work and personal/family life well”, 5-point scale: 1 (strongly disagree) to 5 (strongly agree), α = 0.90 [30]), *employee psychological resilience* (single item, 1(low resilience) to 9 (high resilience), [31]. Participants’ perceptions of their *working environment* was measured using the UK’s Health and Safety Executive’s Management Standards Indicator Tool (MSIT), 35 items using a 5-point scale: 1 (never or strongly disagree) to 5 (always or strongly agree)) [32]) that focuses on six aspects associated with causing stress at work if poorly managed: job *demands* (8 items, example item “I have to work very fast”, α = 0.85)*, control* (6 items, example item “I can decide when to take a break”, α = 0.86)*, support* (from manager and peers, 9 items, example item “I am supported during emotionally demanding work”), α = 0.88)*, relationships* at work (4 items, example item “I Relationships at work are strained”, α = 0.76)*, role* clarity (5 items, “I am clear what is expected of me at work, α = 0.87), and how *change* is managed at work (3 items, example item “Staff are always consulted about change at work”, α = 0.82),. Risks are identified by lower scores on these scales, apart from demands and relationships where higher scores indicate greater risk.

#### 2.1.2. Premenstrual Symptom-Related Variables

Several questions were included to understand premenstrual symptoms and related areas of interest (see Supplementary Table S3). These included the *prevalence* and *severity of premenstrual symptoms* using the 19 item Premenstrual Screening Tool (PSST) [33], which categorizes respondents’ symptom(s) severity into *no/mild*, *moderate (PMS)* or *severe (PMDD)* from responses to 14 premenstrual symptoms and their impact on different life domains using a 4 point scale (1 (not at all) to 4 (severe)). The *duration* of premenstrual symptoms (in days), *how long symptoms had been experienced* (years), and whether participants had *sought professional help* for their premenstrual symptoms in the last 6 months was also asked in the survey. Hormonal contraception use was collected and participants perceived ability to cope with premenstrual symptoms on five premenstrual coping processes measured by the Premenstrual Coping Measure (PMCM) [34]: *avoiding harm* (8 items, example item “I avoid situations that have the potential to provoke me”, α = 0.92)*, awareness-acceptance* of premenstrual change (10 items, example item “I accept my changeable moods”, α = 0.95), *adjusting energy* (5 items, example item “I exercise less”, α = 0.84)*, self-care* (4 items, example item “I allow myself extra time to rest”, α = 0.92), and *communicating* (5 items, example item “I ask for help from others”, α = 0.74) using a 5 point response scale (1 (doesn’t apply to me) to 5 (almost always applies to me)).

#### 2.1.3. Work Outcome Variables

Several work outcomes were examined including (see Supplementary Table S3 for all response options) overall perceptions of *job performance* (5-point scale: 1 (poor), 5 (excellent), [35]), *presenteeism* (the short-form Stanford Presenteeism Scale (SPS-6) [36]) examining participants ability to be fully present at work, concentrate and accomplish tasks despite of premenstrual symptom concerns or difficulties (6 items, example item “Despite having my premenstrual symptoms, I was able to finish hard tasks in my work”, α = 0.78, using a 5-point scale: 1 (strongly disagree) to 5 (strongly agree))*,* the degree of *work absence* because of premenstrual symptoms, and general *turnover intentions*. Specifically, *work absence* was examined by asking if participants had (response options:” yes”, “no”, “sometimes”): (i) *ever any absence* taken because of premenstrual symptoms, (ii) had to *leave work early,* or (iii) *arrived at work late* because of premenstrual symptoms. For those that had taken one of these forms of work absence, (iv) whether this had occurred in the *last four weeks* and if so, the *number of days* affected by absence. *Turnover intentions* were explored in terms of whether participants had considered: (i) *reducing their working hours*, (ii) *leaving the workforce* altogether, and (iii) *leaving their current employing organization* (4 items, example item “How do you feel about leaving this organization? α = 0.83, using a 5-point scale: 1 (definitely not leave), 3 (uncertain), 5 (definitely leave) [37]).

#### 2.1.4. Disclosure, Recommendations and Suggestions for Organizations and Line Managers

With regard to disclosure, participants were asked to indicate (using “yes”, “no”, “sometimes” response options) whether they had (i) *ever disclosed to line managers* about their premenstrual symptoms and work-related difficulties, and, for participants who had taken time off work because of their premenstrual symptoms, (ii) did they disclose this as the reason for their work absence to their line managers. Open-ended questions followed and asked participants to share the reasons for deciding to disclose (or not) to their line managers.

Finally, open-ended response questions were used in the survey to gather data on women’s views on how employing organizations and line managers might better support staff experiencing premenstrual symptom-related difficulties at work. Participants were asked: (i) what they thought would be useful for employers/organizations to do to support staff, and (ii) what line manager behaviors would be helpful and unhelpful.

### 2.2. Analysis

Both quantitative and qualitative analyses were used in this study. Descriptive and inferential analyses are performed on quantitative data to understand the sample and responses overall, as well as to address the specific research questions posed in this paper. For research question 1, descriptive statistical analyses were used to report on the prevalence and severity of premenstrual symptoms in the sample. To address research questions two and three, the moderate and severe premenstrual symptom severity groups were combined to create a dichotomous premenstrual symptom severity outcome variable (no/mild symptoms vs. moderate/severe symptoms) for the subsequent analyses. Pearson Chi-Squared analyses were used and, when required, Fisher’s Exact output was use for interpretation (see [38]). Statistical significance was assumed at the 0.05 level of probability. The statistical software package SPSS (version 25, IBM Corp., Armonk, New York, USA) was used.

The qualitative data from the open-ended responses were analyzed using an inductive thematic analysis approach, following the guidance by Braun and Clarke [39]. The researchers adopted an explicit, surface-level meaning of the data within a realistic epistemological position (i.e., interpretation did not attempt to go beyond the data or words that were provided). The first author [CH] performed the initial analyses and the second author [MSH] reviewed the data and thematic structure to determine if interpretation was agreed. Any differences were discussed and a final set of themes were agreed. Themes were determined using the frequency of codes whereby similar codes were grouped to create a theme. The computer software package NVivo (version 12, QSR International Pty Ltd., Burlington, MA, USA) was used. Analyses were performed separately to develop themes related to thematic structures for responses related for employers and organizations, and for line managers behaviors. The emergent themes between the analyses overlapped considerable and results are discussed together in the results section below.

## 3. Results

### 3.1. Overall Sample Characteristics

One-hundred and twenty-five pre-menopausal women completed the survey and were included in the analysis (one participant had been removed because they were pregnant at the time of completing the survey and therefore had not been experiencing menstrual cycles). They were (see Supplementary Table S1) were, on average, in their late thirties (M = 38.79 years), in a relationship (71.8%), and 90% were heterosexual. Just over half had children to care for (57.3%), and few had other dependents or caring responsibilities (11.3%). Respondents were predominantly white (87.8%), educated to degree level (90.4%), and healthy. Most women were non-smokers (87.2%), took weekly exercise (72.0%), and consumed 1–6 units of alcohol per week or less (76.8%). The average BMI score for the sample was 27.81 (range 17.10–47.20) and within the overweight range [40]. However, 71.2% of the sample rated themselves as having good to excellent general health (M = 2.97, SD = 1.03), 49.6% having good quality of sleep, with mild to moderate levels of anxiety (M = 8.20, SD = 5.85) and depression (M = 7.10, SD = 5.53) [41]. Approximately two-thirds of the sample were using hormonal contraception (67.5%, see Supplementary Table S3).

In terms of their work (see Supplementary Table S2), participants worked an average of 39 h per week (range 15–55 h), were full-time (77.6%), had regular patterns of working hours during the day (87.8%), and almost two thirds could work flexibly (62.6%). The majority had non-manual jobs (98.4%), with no managerial responsibilities (85.4%). On average, the sample were neither satisfied or dissatisfied with their jobs (M = 3.92, SD = 1.47); just under half were satisfied in their jobs (44.7%). However, they did appear to have high levels of job stress (80.6% moderate-severe responses) with a somewhat poor work–life balance (M = 2.55, SD = 0.77) with around a third (30.4%) overall showing agreement work–life balance agreement They also perceived themselves to have some personal resiliency to ‘bounce back’ from a change or setback from work with just over half (55.0%) indicating higher levels. With regard to aspects of their working environment, the responses appeared mixed; average scores suggest the sample had fairly good role clarity (M = 3.73, SD = 0.69), support (M = 3.45, SD = 0.78), relationships (M = 2.71, SD = 0.48), and control over their jobs (M = 3.20, SD = 0.66), but may be experiencing high job demands (M = 3.54, SD = 0.78), and poor experiences of how changed is managed at their workplace (M = 2.46, SD = 0.96).

In terms of the work outcomes (see Supplementary Table S2), on average the sample felt they were performing their jobs well in comparison to others in a similar position with 74% rating themselves as high to excellent. Average scores on presenteeism showed that the sample felt able to overcome premenstrual symptoms in order to carry out their work duties well and be productive (M = 20.88, SD = 5.17) with only 5% scoring below 12 suggesting they did not feel able to overcome their premenstrual symptoms at work. Overall, there were fairly low levels of turnover intentions with a quarter (25.6%) considering leaving the workforce and around a third (31.2%) probably or definitely intending to leave their current employing organization. However, around half (49.6%) had, or were considering, reducing their working hours.

Premenstrual symptom-related work absence was reported by around a quarter of the sample (21.2%); 14.6% had taken time off work due to their symptoms either a full day off, 21.2% have had to leave work early and 16.0% arrived at work late due to their premenstrual symptoms. Of those who had ever taken time off work (*n* = 18), few had done so in the last 4 weeks (11.1%), which lasted either 1 or 2 days in duration. 24.0% of those that had arrived at work late (*n* = 25) had done so in the last 4 weeks, mostly on one occasion/day (66.7%). A third (36.8%) of those that had arrived late (*n* = 19) had done so in the last 4 weeks, mostly one a single occasion/day.

#### 3.1.1. Research Question 1. Premenstrual Symptom Prevalence and Severity

Over 90% of this sample reported that they had experienced at least one premenstrual symptom in the last 4 weeks; of these, 91.2% had experienced physical symptoms such as breast tenderness, headaches, or joint/muscle pain, bloating, weight gain; 84.5% experiencing fatigue/lack of energy; 93.9% experiencing anger or irritability. Symptoms (see Supplementary Table S3) lasted on average around 5 days and ranged between 1–14 days. Respondents in this study had been experiencing them for an average of 15 years (range 1–40 years). Just under a quarter (21.0%) had sought professional help for their premenstrual experiences (see Supplementary Table S3).

In terms of premenstrual symptom severity, just under two-thirds (60.8%) experienced no or mild premenstrual symptom severity whilst around 40% had experienced moderate (34.4%) to severe (4.8%) premenstrual symptom severity in the last 4 weeks, which affected their normal functioning and could potentially receive a diagnosis of PMS and PMDD, respectively.

#### 3.1.2. Research Question 2. Associations between Premenstrual Symptom Severity and Work Outcomes

Female staff experiencing moderate to severe premenstrual symptoms reported significantly lower presenteeism scores compared to those with mild and no premenstrual symptoms, suggesting there were less able to overcome their symptoms and their work performance was being affected. There were no statistically significant group differences for perceived job performance, nor turnover intentions compared to those with no or mild premenstrual symptoms (see Table 1).

Table 2 shows the rates of absence comparing the no/mild premenstrual symptom severity group and those in the moderate to severe severity group. Significant associations were found between severity of premenstrual symptoms and absence levels. The odds ratios suggest that when compared to staff with no/mild premenstrual symptoms, staff with moderate/severe symptoms were 5.20 times more likely to have taken time off (χ^2^(1) = 9.77, *p* < 0.01), 6.27 time more likely to have left work early (χ^2^(1) = 15.42, *p* < 0.001), and 12.40 times more likely to arrive at work late because of their premenstrual experiences (χ^2^(1) = 19.79, *p* < 0.001 (Fisher’s Exact). Work absence in the last 4 weeks contained too few data to perform analyses.

Intentions to reduce working hours were significantly higher in the group with moderate/severe premenstrual symptoms compared to those with no/mild premenstrual symptoms (χ^2^(1) = 4.36, *p* < 0.05). The odds of participants intending to reduce their hours were 2.17 higher in participants with moderate/severe premenstrual symptoms than no/mild symptoms. No statistically significant associations with intentions to leave the current employing organization or the workforce.

#### 3.1.3. Research Question 3. Associations between Premenstrual Symptom Severity and Individual- and Work-Related Variables

With regard to individual-related variables, staff with moderate/severe premenstrual symptoms compared to staff with no/mild symptoms (*n* = 49 vs. *n* = 76) had significantly poorer levels of general health (M = 2.74, SE = 0.13 vs. M = 3.12, SE = 0.12; *t*(123) = 2.06, *p* < 0.05), consumed higher amount of alcohol (M = 2.33, SE = 0.12 vs. M = 1.84, SE = 0.08; *t*(123) = −3.39, *p* < 0.01), had poorer sleep quality (M = 2.82, SE = 0.12 vs. M = 1.84, SE = 0.08; *t*(123) = −2.96, *p* < 0.01), and were feeling more depressed (M = 10.57, SE = 0.85 vs. M = 6.68, SE = 0.61; *t*(123) = −5.04, *p* < 0.001) and anxious (M = 9.94, SE = 0.79 vs. M = 5.28, SE = 0.55; *t*(123) = −3.82, *p* < 0.001). There were significantly fewer participants with moderate to severe premenstrual symptoms using hormonal contraception than not (*n* = 8 vs. *n* = 39, χ^2^(1) = 6.45, *p* < 0.05)) compared to staff with no or mild premenstrual symptoms (*n* = 28 vs. *n* = 44). In addition, those with moderate/severe premenstrual symptoms less frequently used the premenstrual symptom coping strategies of avoiding harm (M = 2.95, SE = 0.12 vs. M = 2.09, SE = 0.11) and adjusting their energy (M = 3.24, SE = 0.12 vs. M = 2.27, SE = 0.12), compared to staff with no/mild symptoms

With regard to work-related variables, participants with moderate/severe premenstrual symptoms compared to staff with no/mild symptoms reported significantly poorer work–life balance (M = 2.19, SE = 0.14 vs. M = 2.78 SE = 0.12; *t*(123) = −1.90, *p* < 0.01) and lower psychological resiliency (M = 5.04, SE = 0.29 vs. M = 6.03, SE = 0.24; *t*(120) = 2.60, *p* < 0.01). In relation to the perceived working environment, participants with moderate/severe premenstrual symptoms (*n* = 49) compared to staff (*n* = 76) with no/mild symptoms reported significantly higher perceived job demands (M = 3.79, SE = 0.10 vs. M = 3.39, SE = 0.09; *t*(123) = −2.89, *p* < 0.01) and less control over their work (M = 3.01, SE = 0.09 vs. M = 3.32, SE = 0.07; *t*(123) = −2.63, *p* < 0.05. There were no other significant between group differences in other individual or work-related variables.

#### 3.1.4. Research Question 4. Line Manager Disclosure for Premenstrual Symptoms and Work Absence

Overall levels of disclosure to line managers about premenstrual symptoms were very low (8.9%, *n* = 11), and occurred more often amongst women with moderate/severe premenstrual symptom severity (*n* = 7) than those with no/mild symptoms (*n* = 4). In particular, less than less than half (44.4%) of participants who had taken some form of work absence (including being late or leaving work early) because of their premenstrual symptoms (*n* = 18) had disclosed that the real reason for their absence was because of their premenstrual symptoms.

Twenty-six women provided responses to the open-ended questions about disclosure. Of these, two women had disclosed their premenstrual symptoms and need for taking work absence to their line manager. The main reason for their disclosure was because of the severity or difficulty of the symptoms, which were very high (e.g., “*Extreme pain. Needing to take pain killers that are so strong I cannot safely drive on them, so I need to get home before the pain is so bad I can’t drive*”).

The majority of responses were from staff who had not disclosed the real reason for absence (*n* = 20) or had only sometimes disclosed the main reason (*n* = 4). Just under half of these participants (45.8%, *n* = 11) said the main reason for not disclosing was because they felt that premenstrual problems were not a valid reason for needing to take sickness absence from work (e.g., “*feeling exhausted because of a menstrual cycle is not viewed as a genuine reason to take time off work, it is part of everyday life*” and “*Felt that was not a valid explanation*”). Some staff felt that they would not be believed or would be dismissed if they said they were having difficult premenstrual problems (e.g., “*I feel I wouldn’t be believed*”). This may relate in some cases to the gender of the line manager (e.g., “*Frequently have male managers who I know would be embarrassed, or who have expressed dismissive views of women on their periods*”), which over a third of these women said was another main reason given for none disclosure of PMS (37.5%, *n* = 9). Respondents felt that men would be too embarrassed (e.g., “*I have a male manager who would probably die of embarrassment*”) and would prefer to disclose to a female manager. Although, this was not universal; there was some mention that female managers may also be unapproachable or unsupportive (e.g., “*Would expect very little sympathy from her*”) because they not have difficult premenstrual symptoms (e.g., “*also had female managers who say, “I manage fine!”*”). These responses suggest that line managers who do not have their own lived experiences of premenstrual symptoms may be influencing these disclosure behaviors.

Another reason given for not disclosing was the nature of the topic being too personal. A quarter of respondents (25.0%, *n* = 6) noted that the topic was perhaps too embarrassing or uncomfortable to discuss with their line manager and will instead give some other health reason (e.g., “*Not comfortable in doing this. Will report headache or other symptoms but not be specific about why*”). Some respondents, again, made reference that this personal “women’s” topic is not something that male colleague experience. As a consequence, they are reluctant to disclose for fear of being perceived as unable to do their job (e.g., “*I think this makes me look weak at work. I need to be able to do my job regardless of what is going on for me as a woman. Particularly as my males colleagues do not experience this*”).

#### 3.1.5. Research Question 5. Recommendations and Suggestions for Employing Organizations and Line Manager Behavior

Fifty-six participants provided suggestions or recommendations for employing organization about supporting staff who may be experiencing difficult premenstrual symptoms at work. Six main recommendations and suggestions were mentioned: showing *understanding and acceptance* (33.3%, *n* = 26), *flexibility* (25.6%, *n* = 20)*,* the *physical work environment* (10.3%, *n* = 8)*, talking* (9.0%, *n* = 7)*, health and wellbeing resources* (7.7%, *n* = 6)*,* and *procedures and policies* (7.7%, *n* = 6). Sixty-two participants provided suggestions and recommendation for line manager behaviors. There were four main themes including (in order of prevalence) *acknowledgment and understanding* (58.0%, *n* = 91), *talking and use of language* (21.0%, *n* = 33), allowing *flexibility and workload* adjustments (17.2%, *n* = 27), and the *work environment and absence management* (3.1%, *n* = 6). These themes show similar and inter-related recommendation and suggestions for organizations and line managers and will therefore be discussed together below.

##### Showing Understanding and Acceptance

Recommendations for employers emphasized the need for understanding of these female health-related experiences and accepting that they happen. This should be done *without judgment* and being *empathic* toward staff who are affected (e.g., “*More knowledge and understanding for line managers. Just because my line manager is fine does not mean I am*”). Respondents believed that female staff should know it is acceptable to be affected by their premenstrual symptoms and to speak-up and ask for support or time off. Demonstrating such *beliefs by* showing support that these women are not fabricating their symptoms and experiences (e.g., “*belief that it is a real problem and not just an excuse women use*”). Employing organizations should be being *mindful* that these premenstrual symptoms and experiences can occur and employers should show staff their *support* when staff experiencing difficulties related to their premenstrual symptoms.

Line managers were given similar suggestions including *acknowledging* these types of staff difficulties in relation to premenstrual symptoms, and being *understanding* and *supportive* towards these staff (e.g., “*Just be understanding*”, “*acknowledge that it affects us*”). Line managers were advised to *be sensitive* towards staff and *show empathy*. Respondents highlighted that it is important for managers to be *non-judgmental* and *trust* what staff are experiencing and disclosing. These premenstrual experiences should be *taken seriously* and *treated like any other health condition*. Conversely, unhelpful behaviors in relation to this theme were expressed as line managers being *dismissive*, *not believing* staff who are experiencing problems, as well as *ignoring* the staff members’ problem as all together (e.g., “*Ignore or be dismissive*”). Not being believed can make the situation worse for some staff (e.g., “*Make you feel worse by not accepting it’s a real issue*”). Showing a *lack of empathy*, *understanding and support* was consistently highlighted as unhelpful behaviors that managers could respond with as it can make the member of staff feel that their experiences are not important and undermine them (e.g., “*Undermine how you’re feeling*”. This includes *making assumptions* and *underestimating* experiences, along with *making staff feel guilty* or that they are *letting others down* because they cannot complete a particular task or objective (e.g., “*Not make you feel like a worthless piece of shit for being a woman with difficult periods. Not make you feel like you’re letting other people down because you’re sick with something that isn’t your fault. Not make you feel guilty if a target is missed because you’re sick and in pain*”).

##### Appropriate Talking and Communication

The importance of talking and communication was raised across responses. Some respondents mentioned that there should be more talking about this issue, including in public that could help raise awareness and facilitate acceptance (e.g., “*make it more spoken about in public*”) as suggested in the above theme. In addition, employers could consider having a policy, which could involve staff training and promote more communication and talking about this health topic (e.g., “*Have a clear policy around this issue which would promote its discussion particularly with male managers who in my experience would struggle to discuss anything linked to women’s reproductive cycles*”).

Suggestions for line managers *communication* and their *use of language* highlighted the importance for line managers to talk openly, in an “adult way” and avoid phrases or words that are considered disrespectful, unhelpful or inappropriate (e.g., “*Talk about it openly and in an adult way, too many people find it gross and/or a skive*”). In addition, line managers should *not make jokes* about premenstrual symptoms and *not use sexist, patronizing, or negative remarks* that might insinuate staff that menstruate is a negative situation (e.g., “*Use words like ‘yuck’ eugh’ ‘that’s gross’ when talking about periods. It’s just blood. Yet so many people get grossed out by it*”). For example, some phrases to be avoided including “*are you premenstrual??”, “we all have to deal with it*”, or “*you seem premenstrual*”.

Conversely, if staff do talk about their premenstrual symptoms and difficulties at work, it is important that line managers *listen* so that they can understand what the member of staff is experiencing and be able to manage the situation in a more tailored/individual and appropriate way (e.g., “*discuss how it affects you so they can be aware of how you’re feeling each month*” and “*asking if they need flexible working for a couple of days*”). Being too *intrusive*, however, should be avoided. Treating the conversations as confidential is also important and not *sharing the member of staff’s experience with others*. Respondents highlighted this to be a common experience by female staff. Conversation should not be avoided but instead handled in a suitably respectful way.

##### Accommodating Policies and Flexibility

The importance of being flexible towards women’s working and having appropriate policies and procedures in place to permit these to happen were recommended (e.g., “*enable flexible working*”). Allowing staff to work flexibly and have more control over their workloads and how they do their tasks was a suggested helpful approach to take. Allowing workloads to be adaptive to a member of staff’s their menstrual cycle could be useful. Respondents suggested that allowing female staff to adjust their work, the hours, and be able to have more flexible working to accommodate the days when they need to not be at work, doing certain tasks, or perhaps, need to take some of the workload off could be very useful for employers and for line managers.

In addition, ensuring workloads and deadlines were not “unrealistic” were also highlighted as something important for employers to ensure (e.g., “*stop giving unreasonable workloads*”). During the premenstrual phase staff may need some temporary allowances or adjustments to their workload. Some women felt that they were being penalized because they were sharing their health difficulties and not being supported in a way they felt they needed. Therefore, having policies in place to allow these members of staff to have temporary adjustments to their work without penalties is encouraged. For example, *not recording days taken off work* because of difficult premenstrual symptoms would be beneficial, as this would avoid the procedure of investigation into why staff are taking time off work to be triggered (e.g., “*Don’t count days taken off with period pain as sick days. Otherwise people end up on sickness procedures over a medical condition they have no control over*”). As premenstrual symptoms are recurring and common, it was considered better to avoid these investigations because staff cannot help experiencing these difficulties but they might result in disciplinary action towards the member of staff for taking too much time off at work. It was acknowledged that female staff should be able to take time off work if they are not well enough to attend and they should not be made to feel guilty or penalized.

##### Appropriate Resources and Work Environments

Offering staff health and wellbeing resources was another suggestion by some respondents (e.g., “*Offer health and wellbeing sessions looking at diet…No one ever seems to make this connection*…*exercise and lifestyles*”), which included having menstrual products available at work, and forms of treatment that could be offered by workplaces or subsidized. Addressing aspects of the work environment were also highlighted. This included both physical aspects of the workplace making sure they are fit for purpose. Suggestions made included making sure toilets had sinks in them, private rooms that were quiet to take a rest, and generally that facilities are considered in relation to these menstrual health issues (e.g., “*consider their buildings with regards to toilets, comfort areas etc., as many tend not to be fit for purpose*”). Non-physical aspects of the work environment were also noted such encouraging a good work–life balance (e.g., “*encourage better work life balance including exercise*”) and promoting a safe space for women at work (e.g., “*Create an environment where women feel they have a voice. I work in a heavily female environment but most of the managers are men*”). Other suggestions included making sure the *physical work environment* was fit for purpose. These included making sure toilets had sinks in them, private rooms that were quiet to take a rest, and generally that facilities are considered in relation to these particular health topics too (e.g., “*consider their buildings with regards to toilets, comfort areas etc., as many tend not to be fit for purpose*”).

## 4. Discussion

This study explored the topic of premenstrual symptom experiences in the work context. We found that premenstrual symptoms were highly prevalent in this UK sample. Around 40% experienced moderate to severe symptoms that impaired normal functioning. This includes 5% with levels of symptom severity that may qualify for diagnosis of PMDD and 35% PMS. These rates align with those found in some other populations previously studied whereby 40% females have symptoms of PMS [42], although slightly lower than the recent global-pooled estimate [2]. The observed prevalence rates severe premenstrual symptoms (and a possible PMDD diagnosis) also concur with previously published rates of PMDD of between 5–8% [7]. However, there are reported variations depending on the methods used to identify and diagnoses PMS and PMDD [8].

Premenstrual symptom severity was associated with several work outcomes. Staff with moderate/severe symptoms were found to be significantly more likely to perceive their premenstrual symptoms as preventing them from being fully present and able to carry out their job (i.e., presenteeism), take time off work because of their premenstrual symptoms, leave early because of their symptoms, and more likely to arrive at work late because of their symptom severity. Previous research has also shown higher rates of work absence in staff with more severe premenstrual symptoms, including partial days off because of their premenstrual symptoms (e.g., [14,15,16,17,18,20]). The importance of considering presenteeism, including staff experiencing difficulty in concentrating during their premenstrual “episode” phase, has been highlighted in previous studies [17,23], which the present study confirmed its importance. A previous study making the impact of PMDD on work highlighted that the long-term impact of working through difficult premenstrual symptoms may be that staff leave the workforce and the careers [17]. The present study also showed that intentions to reduce working hours did not coincide with intentions to leave the employing organization or the workforce. It may therefore only be staff with very high severity and/or having experienced these symptoms over a login period of time where these turnover intentions may occur. Longitudinal research is needed to examine this further. With regard to reducing working hours, implications of this finding suggest that female staff working part-time may be more likely to have moderate/severe symptoms. If so, providing support and work adjustments to address or work around these symptoms may be able to help staff work more hours. Recommendation and suggestions for such support to staff are provided in this study. However, again, longitudinal research would be needed to examine this further.

The present research found no significant differences in job performance. Previous studies have shown lower job performance and productivity levels in staff with more severe premenstrual symptoms compared to those with no/mild severity (e.g., Borenstein and colleagues [13,14]. The different results may relate to differences in the measurements used to calculate job performance and work productivity, or because of the samples investigated who may be from different sectors and/or countries. Future research using objective measures of job performance and output may therefore be useful and offer a way to make more consistent comparisons across different samples.

A number of individual and work-related variables were significantly associated with premenstrual symptom severity. This included levels of general health, alcohol consumption, quality of sleep, levels of depression and anxiety, use of hormonal contraception and using premenstrual coping strategies of avoiding harm and adjusting energy. Aspects of work associated with premenstrual symptom severity included perceived work–life balance, psychological resiliency to change, and perceived job demands and control. Previous research exploring risk factors related to premenstrual symptoms impacting on normal functioning have identified alcohol intake, smoking, weight gain, as well as depression [8] as key factors associated with PMS. Work–life balance was also suggested in a previous qualitative study on staff with PMDD [17]. The other variables found in this study therefore support and build upon previous research to help identify possible risk factors that may be appropriate to consider and address when examining premenstrual symptoms in the work context.

Disclosure of premenstrual symptoms to line managers was very low and occurred more in staff with moderate to severe premenstrual symptoms. Disclosure was significantly higher when women had severe symptoms and needed to be off work. However, those who decided not to disclose revealed it was due to perceiving that premenstrual symptoms would not be seen as a valid reason for needing absence, it would be discriminated, and or cause embarrassment. This was particularly in relation to male line managers but not always as some female managers were also regarded as unapproachable or unsupportive. Some staff felt the issue was too personal and sometimes gave another health reason for taking time off work and were fearful that if they gave the real reason they would be perceived as unable to do their job. These findings support previous research highlighting concerns regarding disclosure of experiencing or taking sickness absence related to problematic premenstrual symptoms [17]. Similar problems around disclosure of health problems can also be found for other health topics, such as mental health [43], being HIV positive [44], or the menopause, which are also often considered a taboo and stigmatized health topic in the work context [22,28,45]. The implications of not disclosure difficulties, or giving another reason for absence, may mean that line managers and employers are unaware of the difficulties some of their staff may be experiencing and in turn, not offer appropriate support and work adjustments.

This study also highlighted several key areas that employers, line managers may be able to address that would help female staff experiencing premenstrual symptoms and difficulties from the perspective of female staff. These centered around the importance of awareness and acceptance of premenstrual symptoms and the potential difficulties that they may temporarily cause for some staff. Being open and facilitating talking and communications about this health topic at work, having policies and practices that can accommodate this health topic, and providing resources and a “fit for purpose” working environment. Specific recommendations and suggestions for employers and line mangers pro-vide clear implications for policy, practice and further research. For example, developing training and resources for organizations and line managers. These suggestions may also be of interest to other key stakeholder such as researchers, policy makers, trade unions, and government bodies that aim to improve the health and wellbeing of the workforce to enhance economic participation. Future research examining the impact of COVID-19 on the working lives of women and how this may influence premenstrual symptom experiences and suggestions for working would also be of value for this topic.

Given that the UK has over 10.5 million women in employment aged between 16–49 years [46] there may be a significant proportion of staff may be working through phases of premenstrual related difficulties on a monthly basis. In particular, staff with moderate to severe premenstrual symptoms may experience a significantly impaired ability to complete their work as normal and avoid distractions at work. They may also be more likely to time off work, especially at the start of their work shift, and consider reducing their working hours. In addition, these issues may be likely to go unknown because of low levels of disclosure of these health difficulties. The research therefore presents timely work as there is growing interest in reproductive health as a public health issue, particularly in the UK [47].

This study has provided new insights into this under-researched health area and directions for future research, policy and practice in organizations. However, limitations of the research include the cross-sectional design of the study, which limits the ability to infer causation, for example, it cannot be ascertained whether premenstrual symptom severity causes the impairments in work outcomes or vice versa. With larger samples and a longitudinal design, it may be possible to control for additional variables (e.g., hormonal contraception use, depression, anxiety) to determine the extent to which work factors may be causing or influencing these premenstrual symptom severity levels and which are the most important to address. In addition, the depth of the qualitative analysis and interpretation was limited by the amount of data provided in the open-text boxes by participants. Other methods of gathering qualitative data, such and interviews and focus groups, would have allowed more in-depth insight into the reasons and explanations given and explore possible relationships between the themes and factors identified here in this study. The results found in this study may therefore serve as a useful guide to inform future research.

## 5. Conclusions

This study examined women’s experience of premenstrual symptoms in the work context. It has shed light on the prevalence and severity of premenstrual symptoms in UK working women, individual and work-related factors associated with symptom severity, association with different work outcomes of interest, levels of disclosure and the reasons behind deciding to disclose (or not), and finally, recommendations and suggestion for organizations and line managers from the female staff perspective. The work highlights the large proportion of women affected by premenstrual experiences, the impact this may have on work and what changes might be helpful for women, managers and organisations.

## Figures and Tables

**Table 1:** Results of independent t-tests between premenstrual symptom severity and job performance, presenteeism, and intention to leave the current employing organization (turnover intention).

Work Outcome	*n*	No/Mild Premenstrual Symptom Severity	*n*	Moderate/Severe Premenstrual Symptom Severity	T-Value	*p*
Job performance	75	M = 3.97 (SD = 0.75)	48	M = 3.75 (SD = 0.73)	1.62	0.12
Presenteeism ^1^	74	M = 22.80 (SD = 4.81)	47	M = 17.87 (SD = 4.21)	5.93	0.00 ***
Turnover intention ^2^	76	M = 3.07 (SD = 1.14)	49	M = 3.17 (SD = 0.86)	−0.53	0.60

^1^ Related to premenstrual symptoms, ^2^ Intention to leave the current employing organization; M = Mean, (SD = standard deviation); *** *p* < 0.001.

**Table 2:** Frequency (n’s) of responses to work absence and intentions to reduce work hours, leave the workforce.

Variable	Response	No/Mild Premenstrual Symptom Severity	Moderate/Severe Premenstrual Symptom Severity
**Work Absence ^1^**:			
(i) Ever taken time off	No	70	35
	Yes	5	13
(ii) Ever left early	No	66	27
	Yes	7	18
(iii)Ever late	No	70	30
	Yes	3	16
**Turnover Intentions:**			
(i) Intention to reduce working hours	No	44	19
	Yes	32	30
(ii) Intention to leave workforce	No	60	33
	Yes	16	16

^1^ Related to premenstrual experiences.

## Data Availability

Restrictions apply to the availability of these data. Data was obtained as part of a invited piece of work by a UK organization and therefore may be sensitive to free distribution and use. The data may be available upon request to the corresponding author and with permission of the organization from whom the data were collected.

## Supplementary Materials

The following are available online at https://www.mdpi.com/article/10.3390/ijerph18073647/s1, Table S1: Sample responses for the individual-related variables (*N* = 125), Table S2: Sample responses for the work-related variables and work outcomes (*N* = 125), Table S3: Sample responses for the premenstrual-related variables (*N* = 125).
