# Supplementary material for: Premenstrual Symptoms and Work: Exploring Female Staff Experiences and Recommendations for Workplaces
Source: Int J Environ Res Public Health. 2021 Mar 31;18(7):3647. doi: 10.3390/ijerph18073647 (PMC8036722; doi:10.3390/ijerph18073647)
Supplement: Supplementary file 1 [file ijerph-18-03647-s001.pdf]

Supplementary Material

Table S1. Sample responses for the individual-related variables (N = 125).

Variable		Means (SD), % (*N*s)
Age (years) (*n* = 119)		M = 38.79 (SD = 6.35) (range 24–54)
Ethnicity (*n* = 124)	White (British, Irish, European, Other)	87.9% (*n* = 111)
	Black (Caribbean, Other) / Asian (Indian, British, Other)	5.6% (*n* = 7) / 4.8% (*n* = 6)
Educational level	≤ Higher/A-level/National Grade	8.8% (*n* = 11)
	Degree/Professional or Postgraduate	90.4% (*n* = 113)
Sexuality	Heterosexual	89.6% (*n* = 112)
	Bisexual / Lesbian	4.0% (*n* = 5) / 3.2% (*n* = 4)
Relationship status (*n* = 124)	Single/ Divorced/ Separated	28.2% (*n* = 35)
	In a relationship /co-habiting/ married	71.8% (*n* = 89)
Dependents (*n* = 124)	Children	57.3% (*n* = 71)
	Friend/relative/other	11.3% (*n* = 14)
General health	1 (poor)	10.4% (*n* = 33)
	2 (fair)	18.4% (*n* = 23)
	3 (good)	40.0% (*n* = 50)
	4 (very good)	26.4% (*n* = 33)
	5 (excellent)	4.8% (*n* = 6)
		M = 2.97 (SD = 1.03)
BMI (*n* = 117)		M = 27.81 (SD = 6.46) (range 17.10–47.20)
Exercise frequency	1 (rarely/never)	16.0% (*n* = 20)
	2 (less than once per week)	12.0% (*n* = 15)
	3 (once per week)	16.0% (*n* = 20)
	4 (2-3 times per week)	36.8% (*n* = 46)
	5 (4-6 times per week)	15.2% (*n* = 19)
	6 (every day)	4.0% (*n* = 5)
		M = 3.35 (SD = 1.41)
Alcohol consumption (units per week)	1 (none)	25.6% (*n* = 32)
	2 (1-6 units per week)	51.2% (*n* = 64)
	3 (7-13 units per week)	17.6% (*n* = 22)
	4 (+14 units per week)	5.6% (*n* = 7)
		M = 2.03 (SD = 0.81)
Smoker status:	Never smoked	47.2% (*n* = 59)
	Ex-smoker / Smoker	40.0% (*n* = 50) / 12.8% (*n* = 16)
Sleep quality	1 (very good),	5.6% (*n* = 7)
	2 (fairly good),	44.0% (*n* = 55)
	3 (fairly bad),	38.4% (*n* = 48)
	4 (very bad)	12.0% (*n* = 15)
		M = 2.57 (SD = 0.78)
Depression (GAD-7) [a]		M = 7.10 (SD = 5.53) (range 1–21)
Anxiety (PHQ-9) [a]		M = 8.20 (SD = 5.85) (range 1–27)

[a] Total summed score.

Table S2. Sample responses for the work-related variables and work outcomes (N = 125).

Variable	Response Options/Sub-Scale	Means (SD), % (*N*s)
Working hours (per week) (*n* = 120)		M = 38.83 (SD = 8.79) (range 15–55)
Work status	Full-time	77.6% (*n* = 97)
	Part-time	22.4% (*n* = 28)
Work pattern/shift work (*n* = 123)	Regular (day)	87.8% (*n* = 108)
	Regular (night)	0.8% (*n* = 1)
	Set hours (day/night)	4.1% (*n* = 5)
	No set hours/pattern	5.7% (*n* = 7)
	Other	1.6% (*n* = 2)
Flexible working(*n* = 123)	Yes	62.6% (*n* = 77)
	No	37.4% (*n* = 46)
Type of work (*n* = 124)	Non-manual	98.4% (*n* = 122)

	Manual / Both	0.8% (*n* = 1)/ 0.8% (*n* = 1)
Managerial/supervisory responsibilities (*n* = 123)	Yes	14.6% (*n* = 18)
	No	85.4% (*n* = 105)
Sector	Public	68.8% (*n* = 86)
	Private	31.2% (*n* = 39)
Job satisfaction	1 (extremely dissatisfied)	6.4% (*n* = 8)
	2 (very dissatisfied)	10.4% (*n* = 13)
	3 (dissatisfied)	27.2% (*n* = 34)
	4 (neither dissatisfied or satisfied)	11.2% (*n* = 14)
	5 (satisfied)	31.1% (*n* = 39)
	6 (very satisfied)	12.8% (*n* = 16)
	7(extremely satisfied)	0.8 (*n* = 1)
		M = 3.92 (SD = 1.47)
Job stress (*n* = 124)	1 (not stressful)	3.2% (*n* = 4) (Mild)
	2	16.1% (*n* = 20) (Mild)
	3	50.0% (*n* = 62) (Moderate)
	4 (extremely stressful)	30.6% (*n* = 38) (Severe)
	*This was re-coded to become a 3-point scale mild (1)-severe (3)	M = 3.08 (SD = 0.77)
Work-life balance		M = 2.55 (SD = 1.08)
Employee psychological resilience (*n* = 122)	1(low resilience)	3.3% (*n* = 4)
	2	5.7% (*n* = 7)
	3	8.2% (*n* = 10)
	4	10.7% (*n* = 13)
	5	17.2% (*n* = 21)
	6	14.8 (*n* = 18)
	7	19.7% (*n* = 24)
	8	13.9% (*n* = 17)
	9 (high resilience)	6.6% (*n* = 8)
		M = 5.65 (SD = 2.08)
Working environment:	Demands	M = 3.54 (SD = 0.78)
	Control	M = 3.20 (SD = 0.66)
	Support	M = 3.45 (SD = 0.78)
	Relationships	M = 2.71 (SD = 0.48)
	Role	M = 3.73 (SD = 0.69)
	Change	M = 2.46 (SD = 0.96)
Work outcomes:		
Job performance (*n* = 123)	1 (poor)	0% (*n* = 0)
	2	4.1% (*n* = 5) (Low)
	3	22.0% (*n* = 27) (Medium)
	4	55.3% (*n* = 68) (High)
	5 (excellent)	18.7% (*n* = 23) (Excellent)
	*This was re-coded to become a 4-point scale low (1)-excellent (4)	M = 3.89 (SD = 0.75)
Presenteeism[a] (*n* = 121)		M = 20.88 (SD = 5.17) (range 9–30)
Work absence due to premenstrual symptoms:		
Ever taken time off (*n* = 123)	Yes:	14.6% (*n* = 18)
	Last 4 weeks (*n* = 18)	11.1% (*n* = 2)
	Days in last 4 weeks (*n* = 2)	M = 1.50 (days)
	No	85.4% (*n* = 105)
Ever left early (*n* = 118)	Yes:	21.2% (*n* = 25)
	Last 4 weeks (*n* = 25)	24.0% (*n* = 6)
	Days in last 4 weeks (*n* = 6)	M = 1.33 (days)
	No	78.8% (*n* = 93)
Ever late (*n* = 119)	Yes:	16.0% (*n* = 19)
	Last 4 weeks (*n* = 19)	36.8% (*n* = 7)
	Days in last 4 weeks (*n* = 7)	M = 1.14 (days)
	No	84.0% (*n* = 100)
Turnover intentions:		
Intention to reduce working hours	Yes / Sometimes	36.0% (*n* = 45)/ 13.6% (*n* = 17)

	No	50.4% (*n* = 63)
Intention leave workforce	Yes / Sometimes	15.2% (*n* = 19)/ 10.4% (*n* = 13)
	No	74.4% (*n* = 93)
Intention to leave current employing organization		M = 3.11 (SD = 1.04)

[a] Total summed score.

Table S3. Sample responses for the premenstrual-related variables (*N* = 125).

**Variables**		**Means (SD), % (*N*s)**
Premenstrual symptom severity:	No or mild symptoms	60.8% (*n* = 76)
	Moderate symptoms / Severe symptoms	34.4% (*n* = 43) / 4.8% (*n* = 6)
Duration of premenstrual symptoms:	Days (*n* = 121)	M = 4.59 (SD = 3.25) (range 1–14)
	Years (*n* = 115)	M = 15.37 (SD = 11.36) (range 1–40)
Sought professional help in last 6 months: (*n* = 124)	Yes	21.0% (*n* = 26)
Hormonal contraception use: (*n* = 123)	Yes	67.5% (*n* = 83)
Premenstrual symptom coping: (*n* = 124)	Avoiding harm	M = 2.43 (SD = 0.98)
	Awareness-acceptance	M = 3.31 (SD = 0.95)
	Adjusting energy	M = 2.65 (SD = 1.04)
	Self-care	M = 2.45 (SD = 1.01)
	Communicating	M = 2.26 (SD = 0.80)
